# Prognostic Value of PLXND1 and TGF-β1 Coexpression and Its Correlation With Immune Infiltrates in Hepatocellular Carcinoma

**DOI:** 10.3389/fonc.2020.604131

**Published:** 2021-01-08

**Authors:** Juanni Li, Kuan Hu, Dongren He, Lei Zhou, Zhiming Wang, Yiming Tao

**Affiliations:** ^1^ Department of Pathology, Xiangya Hospital, Central South University, Changsha, China; ^2^ Department of General Surgery, Xiangya Hospital, Central South University, Changsha, China; ^3^ Department of Anesthesiology, Third Xiangya Hospital of Central South University, Changsha, China

**Keywords:** plexin D1, hepatocellular carcinoma, tumor immune microenvironment, prognosis, transforming growth factor-beta

## Abstract

Hepatocellular carcinoma (HCC) is the most common primary liver malignancy with no curative treatments. Plexin D1 (PLXND1) is a cellular receptor whose functions have been explored in several human cancers; however, the critical roles of PLXND1 in HCC have rarely been probed. Therefore, the present study attempted to elucidate the expression pattern, prognostic significance, and potential roles of PLXND1 in HCC. We found that PLXND1 expression in HCC tissues was significantly higher compared with normal liver tissue from Gene Expression Profiling Interactive Analysis (GEPIA) and Integrative Molecular Database of Hepatocellular Carcinoma (HCCDB) databases. This result was further validated by immunohistochemistry staining (IHC) using tissue microarrays, which contained 216 HCC cases collected from our hospital. Additionally, PLXND1 expression showed a significant correlation with several clinical characteristics, including tumor grade and tumor hemorrhage (TH). Moreover, TISIDB and GEPIA databases were used to investigate the roles of PLXND1 in tumor-immune system interactions in HCC. As an immunoinhibitor, transforming growth factor-beta (TGF-β1) displayed the greatest correlations with PLXND1 in HCC. Finally, Kaplan-Meier curves and Cox analysis were conducted to further examine the potential clinical value of PLXND1 in HCC. We described a subclassification of HCC based on PLXND1 and TGF-β1 expression, which could be used to predict clinical outcomes and patient prognosis. Taken together, the results of this study indicate that PLXND1 might be a promising prognostic biomarker and potential therapeutic target in HCC.

## Introduction

Hepatocellular carcinoma (HCC) is the most common primary liver malignancy and the fourth leading cause of cancer death worldwide (1–3). More than 70% of patients are diagnosed at an advanced stage with a dismal prognosis. Surgical resection and chemotherapy are the conventional treatment for HCC, but the mortality rate of this cancer remains high (4). In recent years, immunotherapy is increasingly considered as one of the most promising approaches for identifying drug targets for therapeutic interventions (5, 6). The precise estimation of patient prognosis plays an important and irreplaceable role on the treatment decision of HCC patients. Hence, the exploration and identification of novel therapeutic biomarkers and reliable prognostic biomarkers are urgently required.

Plexins are a family of transmembrane proteins that serve as cellular receptors for semaphorins and have a range of critical functions in vascular patterning, axonal guidance, cell proliferation, migration, and immune cell regulation (7–9). Plexin D1 (PLXND1), a newly identified member of the plexin family of molecules, has been reported to be dysregulated in several cancers (7, 10). Emerging data have shown that PLXND1 could function as a tumor suppressor or a classical oncogene. Serving as a tumor suppressor, PLXND1 was reported to promote apoptosis in the absence of semaphorin 3E in breast cancer (10). Conversely, as an oncogene, PLXND1 could promote tumor development and progression by aiding in tumor metastasis and epithelial mesenchymal transition (EMT) (7, 11, 12). In addition, PLXND1 was found to be highly correlated with tumor hemorrhage (TH) in this study and plays a critical role in the tumor immune microenvironment (13, 14). Mumblat Yelena *et al*. showed that PLXND1 was involved in inducing the collapse of the cytoskeleton of lymphatic endothelial cells (LEC), and impacted tumor lymphangiogenesis and metastasis (13): however, until recently, little was known about the abnormal expression and function of PLXND1 in HCC. Thus, the detailed function of PLXND1 in the tumorigenesis and immune infiltration of HCC warrants further investigation.

Due to high mortality rate, limited treatment effects and poor prognosis of HCC, a novel therapeutic strategy with different mechanisms from conventional treatments are urgently needed (15). Immunotherapy has recently emerged as one of the most promising frontiers of cancer treatment, especially the development of immune checkpoint inhibitors, and has yielded promising therapeutic outcomes in many types of solid tumors, including HCC (16, 17). In spite of these advances, fundamental differences in patient responsiveness to these treatments are inexplicable (18). Hence, comprehensive research on the immune microenvironment of HCC needs to be further investigated.

Prior research on PLXND1 in cancer is limited to cancer types apart from liver cancer. For liver cancer, neither abnormal PLXND1 expression nor its correlation with the clinicopathological parameters of HCC have been reported. In this study, we first reported a higher expression of PLXND1 in HCC and its correlation with clinicopathological features. Additionally, PLXND1 expression was related with tumor-infiltrating lymphocytes (TILs) and immunoinhibitors, and TGF-β1 displayed the greatest correlations with PLXND1 expression in HCC. Then, Kaplan-Meier curves and Cox analysis were conducted to further examine the potential clinical significance of PLXND1 in HCC. Taken together, these results highlight the critical roles of PLXND1 in tumorigenesis, the tumor immune microenvironment, and clinical opportunities for cancer research.

## Materials and Methods

### Patient Samples

Written informed consent was obtained from all recruited patients prior to the study. All procedures in this study were approved by the Ethics Review Committee of Xiangya Hospital of Central South University (N201303033). The 216 paraffin-embedded archived liver cancer tissues used in this study were histopathologically and clinically diagnosed at the Xiangya Hospital between 2011 and 2013. No patients in this study received chemotherapy before surgery.

### Immunohistochemistry

IHC staining was performed on tissue microarrays as previously described (19). The following antibodies were used: anti–PLXND1 (ab28762; Abcam, Cambridge, MA) and anti–TGF-β1 (ab25121; Abcam, Cambridge, MA). Expression of PLXND1 were semiquantitatively classified according to the immunoreactive H-score (HS; range 0–300). Subsequently, stratification scoring was conducted according to H-score as follow: HS < 80, scored as 0; 80 < HS <120, scored as 1; 120 < HS < 200, scored as 2; HS > 200, scored as 3. If the score was equal to or greater than 2, the tumor was considered to have high PLXND1 expression; otherwise, low PLXND1 expression was classified. The expression levels of target proteins in tissue were examined by two independent pathologists blinded to the clinical characteristics of the patients.

### Bioinformatics Analysis

Gene Expression Profiling Interactive Analysis (GEPIA, http://gepia.cancer-pku.cn/) is an interactive web server for cancer and normal gene expression profiling and interactive analyses (20). In this database, we compared the PLXND1 mRNA expression between tumors and their paired normal tissues. Additionally, GEPIA enabled us to analyze the link between PLXND1 expression and gene markers of different types of tumor infiltrating immune cells.

Integrative Molecular Database of Hepatocellular Carcinoma (HCCDB, http://lifeome.net/database/hccdb) was developed at Tsinghua University and was used to verify the repeatability of identification of HCC gene expression patterns and the annotation of transcripts (21). We use this tool to analyze the expression of PLXND1.

TISIDB (http://cis.hku.hk/TISIDB) is an integrated repository portal which integrates multiple types of data resources in oncoimmunology (22). By using the multidimensional profiling data for 30 cancer types from TCGA, TISIDB pre-calculated the relation between the immune features (e.g., lymphocytes, immunomodulator) and expression, copy number, methylation, and mutation of any gene. Therefore, users can check “lymphocyte,” “immunomodulator,” or “chemokine” tab to evaluate whether the selected gene might regulate immune features across cancers. The heatmap graphic outputs which show Spearman correlations between expression of the selected genes and immune features across human cancers are implemented with the heatmap module of ECharts (https://echarts.apache.org/). Visualization scatter plot for a Spearman correlation between the selected genes and a specific member of immune features in a specific cancer type can be generated through geom_point function of R package ggplot (R script, https://www.r-project.org/). In this database, we analyzed the correlation between PLXND1 expression and TILs and immunoinhibitors in HCC.

### Statistical Analysis

The statistical analyses were performed with SPSS 23.0 for Mac (SPSS Inc., Chicago, IL). Quantitative values are presented as the mean ± SEM. Paired t tests and Student’s tests were used for paired and unpaired continuous data, respectively. The χ2 test was used for categorical data. The Mann-Whitney test was applied to analyze correlations between PLXND1 expression and clinicopathologic characteristics in HCC. Survival analysis was performed by Kaplan–Meier analysis and log-rank test. A cox proportional hazards regression model was used for univariate and multivariate survival analyses. Significant changes were indicated with asterisks: *= (*P* < 0.05); **= (*P* < 0.01); *** = (*P* < 0.001).

## Results

### Elevated Expression of PLXND1 Messenger RNA in Hepatocellular Carcinoma Tissues

**Figure 1:**
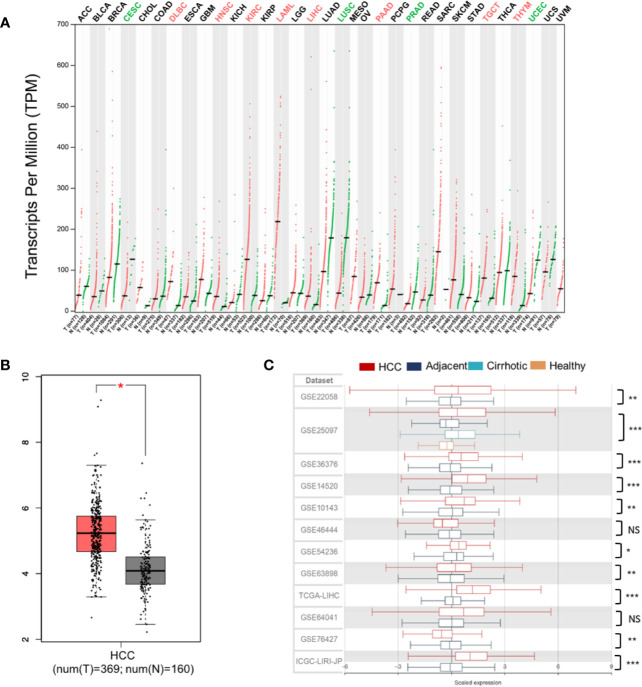
PLXND1 mRNA expression is elevated in hepatocellular carcinoma (HCC). **(A)** The expression of PLXND1 varied among different tissues and tumors across TCGA cancer types from Gene Expression Profiling Interactive Analysis (GEPIA), and the difference was significant in liver hepatocellular carcinoma (LIHC). **(B)** PLXND1 expression in HCC tissues was greater than normal tissues from GEPIA. **(C)** PLXND1 expression in 10 out of 12 clinical cohorts was significantly elevated in HCC tissues compared to the adjacent tissues. Twelve clinical cohorts contain both HCCs and the adjacent normal liver tissues. Only GSE25097 dataset contains tissues (tumor and adjacent non-tumor) from HCCs plus cirrhotic tissues and normal liver tissues. HCC, hepatocellular carcinoma; *P < 0.05, **P < 0.01 and ***P < 0.001; NS, not significant.

For liver cancer, neither abnormal PLXND1 expression nor its association with clinicopathological parameters of HCC has been reported. Here, we investigated the differential expression of PLXND1 using the GEPIA and HCCDB databases. In GEPIA, PLXND1 mRNA expression between tumors and their paired normal tissues across 32 TCGA cancer types were compared. Significantly differential expression was found in 12 cancer types (**Figure 1A**), and elevated expression of PLXND1 mRNA was found in HCC compared with normal liver tissues (**Figures 1A, B**). Next, in HCCDB, we validated PLXND1 mRNA expression in the large cohorts of HCC patients. Compared with the adjacent normal liver tissues, significantly upregulated PLXND1 expression in HCC was found in 10 out of 12 clinical cohorts, suggesting that the upregulation of PLXND1 is mainly attributed to the transformation of normal hepatocytes to cancer cells (**Figure 1C**, **Figure S1**). Moreover, PLXND1 was expressed in various tissues, but its expression was low in normal liver tissues (**Figure S2**).

### PLXND1 Protein Expression and Its Association With Clinicopathological Features

**Figure 2:**
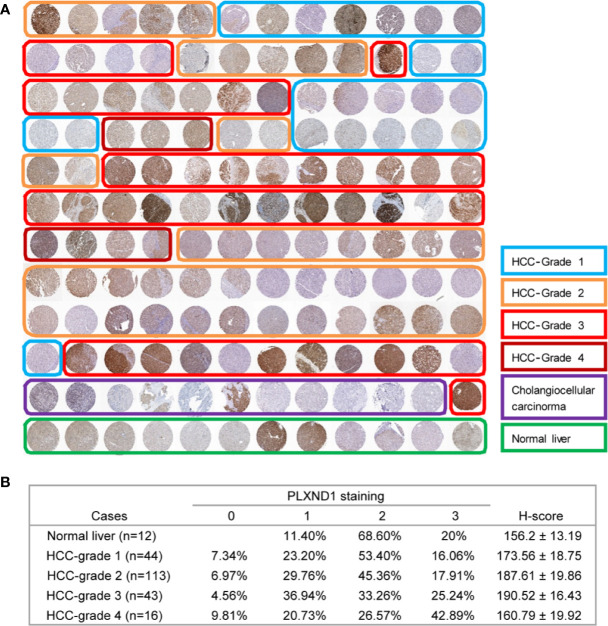
The expression of PLXND1 protein in hepatocellular carcinoma (HCC). **(A)** Tissue microarrays of HCC tumor tissues as well as normal liver tissues were stained for PLXND1 protein. **(B)** PLXND1 signal intensities in normal liver tissues *versus* different grades of HCC tissues were quantified by H-scores, H-score mean ± SD was showed. Blue square, well-differentiated HCC (grade 1); orange square, moderately differentiated HCC (grade 2); red square, poorly differentiated HCC (grade 3); purple square, undifferentiated HCC (grade 4); green square, normal liver. The normal liver tissues were the adjacent normal liver tissues from hepatic hemangioma and distributed in the bottom row of tissue microarray. The HCC tumor tissues were randomly distributed in the upper part of tissue microarray. All these tissues were embedded in the same paraffin block.

To verify the overexpression of PLXND1 protein in HCC, we explored the protein expression level of PLXND1 in HCC by immunohistochemistry staining (IHC) using tissue microarrays and analyzed its associations with clinicopathological variables. Representative images of PLXND1 IHC staining are shown in **Figure 2A**. In the clinical samples, significantly higher PLXND1 expression was found in higher-grade HCC samples (**Figure 2**), which was consistent with the HCC samples from the TCGA database (**Figure S3**). In addition, PLXND1 immunoreactivity was also found to be significantly associated with stage, histology grade, TH, tumor number, and satellite nodules, suggesting the potential oncogenic activity of PLXND1 in HCC (**Table 1**).

**Table 1:** Correlation of PLXND1 expression with clinicopathological characteristics in HCC (n = 216).

Characteristics	PLXND1 [*No*. (%)]		*P*-value
	Low expression	High expression	
**Age**			
≤60 years	26(25.0)	78(75.0)	0.146
>60 years	19(17.0)	93(83.0)	
**Gender**			
Male	37(19.4)	154(80.6)	0.144
Female	8(32.0)	17(68.0)	
**Stage (TNM)**			
I-II	39(24.5)	120(75.5)	0.026
III	6(10.5)	51(89.5)	
**Histology grade** (Edmondson-Steiner)			
I-II	34(21.7)	123(78.3)	0.003
III-IV	11(18.6)	48(81.4)	
**Tumor hemorrhage (TH)**			
Absent	33(33.0)	67(67.0)	<0.001
Present	12(10.3)	104(89.7)	
**Liver cirrhosis**			
Absent	7(35.0)	13(65.0)	0.101
Present	38(19.4)	158(80.6)	
**Tumor size**			
≤5 cm	22(25.9)	63(74.1)	0.141
>5 cm	23(17.6)	108 (82.4)	
**Tumor number**			
Single	35(29.9)	82(70.1)	<0.001
Multiple	10(10.1)	89(89.9)	
**Tumor encapsulation**			
Complete	25(22.7)	85(77.3)	0.485
None	20(18.9)	86(81.1)	
**Satellite nodules**			
Absent	28(29.2)	68(70.8)	0.007
Present	17(14.2)	103(85.8)	

Significant P values are underlined. Mann-Whitney U test was used for comparison between two groups.

### The Regulation of Immune Molecules by PLXND1

**Figure 3:**
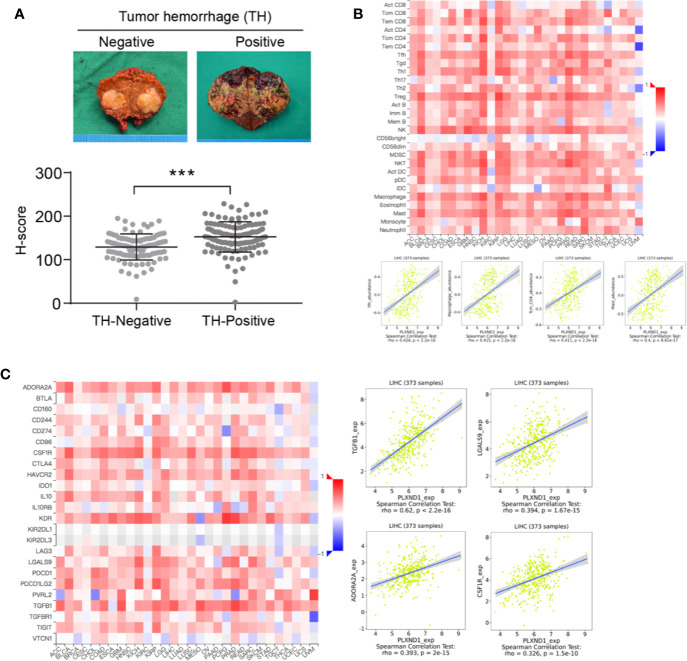
The correlation of PLXND1 expression with tumor hemorrhage (TH), lymphocytes, and immunoinhibitors in hepatocellular carcinoma (HCC). **(A)** Increased expression of PLXND1 protein was found in TH-positive HCC compared with to TH-negative HCC. Representative gross specimens of HCC with/without TH were also presented. **(B)** Upper panel: the correlation between PLXND1 expression and tumor-infiltrating lymphocytes (TILs) in pan-cancer; lower panel: in HCC, four TILs including Tfh cells, macrophages, Tcm_CD4 cells, and mast cells showed a kind of medium correlation with PLXND1 expression. **(C)** Left panel: the correlation between PLXND1 expression and immunoinhibitors in pan-cancer; right panel: in HCC, four immunoinhibitors was positively correlated with PLXND1 expression. TGF-β1 showed a relative strong correlation with PLXND1 expression. ***P < 0.001.

Based on the tumor hemorrhage (TH) status, we classified a cohort of patients with HCC into two subgroups, TH-negative and TH-positive. HCC tumors with TH showed even higher expression of PLXND1 as determined by the H-scores (**Figure 3A**). Our previous study reported that TH indicates a worse tumor immunological microenvironment, especially in HCC (23, 24). Thus, we further explored whether PLXND1 is a significant factor that relates to immune infiltration using the TISIDB and GEPIA databases. In TISIDB, we found that PLXND1 expression was correlated with several tumor-infiltrating lymphocytes (TILs) in HCC patients. Lymphocytes showing the greatest correlations included T follicular helper cells (Tfh; Spearman: r = 0.424, *P* < 2.2e−16), macrophages (Spearman: r = 0.415, *P* < 2.2e−16), central memory CD4 T cells (Tcm_CD4; Spearman: r = 0.411, *P* < 2.2e−16), and mast cells (Spearman: r = 0.4, *P* = 8.62e−17) (**Figure 3B**). We also investigated the correlation between PLXND1 expression and gene markers of different types of TILs, and the results showed that TGF-β1, as a marker of Tregs, was moderately associated with PLXND1 (**Table S1**). Moreover, as shown in **Figure 3C**, PLXND1 expression was associated with several immunoinhibitors in HCC. TGF-β1, a multifunctional cytokine involved in tumor invasion and progression, displayed the greatest correlations with PLXND1 expression in HCC (Spearman: r = 0.62, *P* < 2.2e−16). Therefore, these findings suggest that PLXND1 might exert a significant effect on immune fingerprinting in HCC.

### The Prognostic Value of PLXND1 and TGF-β1 in Patients With Hepatocellular Carcinoma

**Figure 4:**
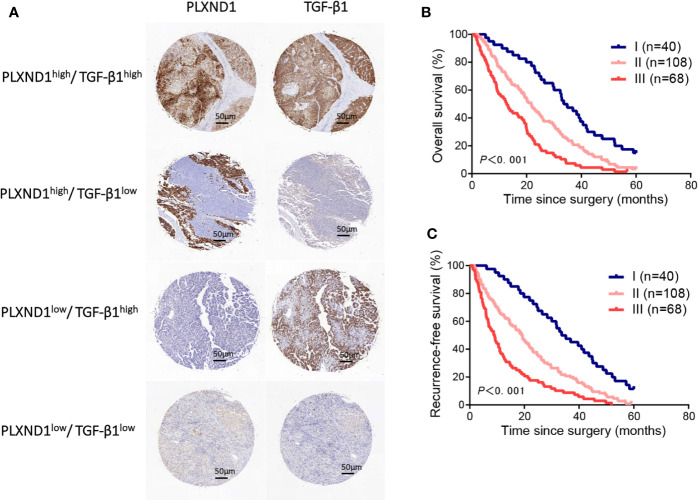
The prognostic value of PLXND1 and TGF-β1 in patients with hepatocellular carcinoma (HCC). Patient tumor tissues were immunostained for PLXND1 or TGF-β1 and classified into three groups: group I, double negative (PLXND1^low^/TGF-β1^low^); group II, PLXND1^high^/TGF-β1^low^ and PLXND1^low^/TGF-β1^high^; and group III, double positive (PLXND1^high^/TGF-β1^high^). **(A)** Representative immunohistochemical staining characteristics of PLXND1 and TGF-β1 expression in each patient group. **(B, C)** Kaplan-Meier analysis of overall survival (OS) and recurrence-free survival (RFS) based on the expression of PLXND1 and TGF-β1 showed that the combination of PLXND1^high^ and TGF-β1^high^ indicated a worse clinical outcome for patients with HCC. High expression: the score was 2 or 3; low expression: the score was 1 or 0.

**Figure 5:**
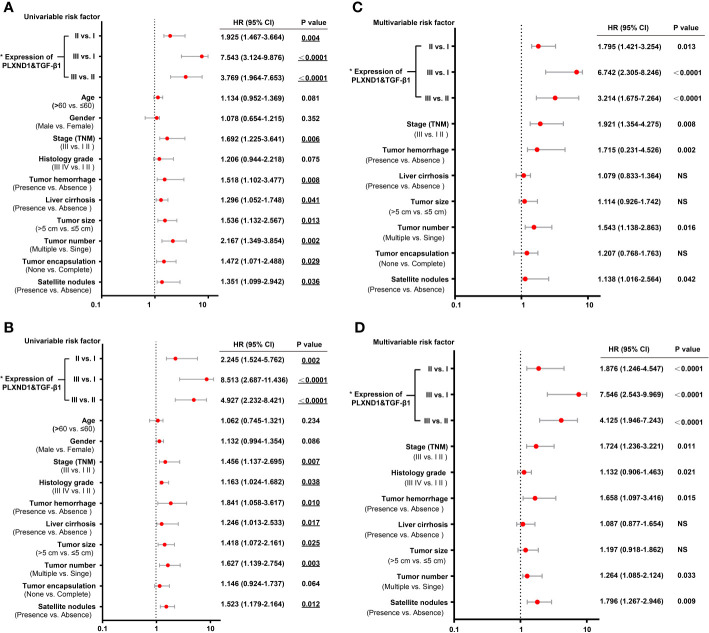
Univariate and multivariate analyses of prognostic factors with overall survival (OS) and recurrence-free survival (RFS) in hepatocellular carcinoma (HCC) patients. **(A)** The univariate analyses of prognostic factors with patient overall survival (OS) are presented in the forest plot. **(B)** The univariate analyses of prognostic factors with patient recurrence-free survival (RFS) are presented in the forest plot. **(C)** The multivariate analyses of prognostic factors with patient OS are presented in the forest plot. **(D)** The multivariate analyses of prognostic factors with patient RFS are presented in the forest plot. The expression of PLXND1 & TGF-β1 are indicated as follows: I, PLXND1^low^/TGF-β1^low^; II, PLXND1^high^/TGF-β1^low^ and PLXND1^low^/TGF-β1^high^; and III, PLXND1^high^/TGF-β1^high^.

We have found that in HCC, PLXND1 expression was correlated with TGF-β1 (**Figure 3C**, **Table S1**), which is a well-known modulator involved in tumor formation, invasion, and metastasis. Here, we further explored whether the coexpression of PLXND1 and TGF-β1 was associated with patient prognosis. We quantified the expression of PLXND1 and TGF-β1 in HCC tissues by IHC, and a varied expression pattern and intensity among the HCC tissues was observed. Representative images of PLXND1 and TGF-β1 IHC staining are shown in **Figure 4A**. We then grouped patients into 3 categories: group I, double negative (PLXND1^neg^/TGF-β1^neg^); group II, PLXND1^pos^/TGF-β1^neg^ and PLXND1^neg^/TGF-β1^pos^; and group III, double positive (PLXND1^pos^/TGF-β1^pos^). Survival analysis showed that the overall survival (OS) rates in group III were significantly lower than those in group II and group I (**Figure 4B**). A consistent result was obtained for the recurrence-free survival (RFS) analysis (**Figure 4C**). In addition, univariate analysis revealed the following features as prognostic factors related with both OS and RFS: the expression of PLXND1 and TGF-β1, stage, TH, liver cirrhosis, tumor size, tumor number, and satellite nodules (**Figures 5A, B**). Multivariate analysis further identified that only the expression of PLXND1 and TGF-β1, stage, TH, tumor number, and satellite nodules behaved as independent predictors for both OS and RFS (**Figures 5C, D**).

## Discussion

In this study, we aimed to investigate critical and novel biomarkers involved in the development and progression of HCC. PLXND1 belongs to a family of single-pass transmembrane proteins called plexins and serves as a receptor for semaphorins (25–27). Semaphorin-plexin signaling is known to be essential for many cellular aspects of organogenesis, including cell proliferation, invasion, and survival (28, 29). Emerging data have shown that the expression of PLXND1 is often dysregulated in several types of cancer and acts either as a tumor suppressor or an oncogene. Some studies have shown that PLXND1 was overexpressed in tumor cells and promoted tumor development and progression in several cancers, including colon cancers (11), melanoma (30), pancreatic cancer (31), and ovarian cancer (12). Conversely, J. Luchino, M. et al. revealed that PLXND1 signaling could have antitumor activity as well and stimulated cell apoptosis in breast cancer (10): however, the detailed roles of PLXND1 in HCC have rarely been studied. In this study, we first revealed an abnormal expression level, prognostic value, and potential roles of PLXND1 in HCC biology. We found that PLXND1 expression in HCC tissues was significantly higher compared to normal liver tissues using data from the GEPIA and HCCDB databases. This result was further validated by IHC using tissue microarrays, which contained 216 HCC cases collected from our hospital. A high expression of PLXND1 was significantly correlated with several markers of HCC progression, including tumor grade and tumor hemorrhage (TH). These data suggest that PLXNDA may be a potential biomarker and therapeutic target in HCC.

Recently, increasing evidence has shown that the tumor microenvironment and immune infiltration plays a critical role in tumor development and progression (32–34). Our previous study revealed that tumor hemorrhage (TH) indicated a worse tumor immunological microenvironment, especially in HCC (23, 24). In this study, PLXND1 was found to be highly correlated with TH in HCC. These above findings inspired us to further investigate the correlation between PLXND1 and immunity in HCC development. We assessed the correlation between PLXND1 and the immune system using the TISIDB and GEPIA databases. The results showed that PLXND1 had the most significant correlation with tumor-infiltrating lymphocytes including Tfh cells, macrophages, Tcm_CD4 cells, and mast cells. Tfh cells are a subset of CD4+ helper T cells that play a crucial role in humoral immunity and protection against pathogens and in the generation of autoimmunity (32, 35). Macrophages, as the forefront of host defense, also play pivotal roles in tumor immunity (36, 37). Mast cells consistently infiltrate tumors and can be both a driving or an opposite force for tumor progression depending on the tumor microenvironment (38, 39). Additionally, TGF-β1 was revealed to be the most likely immune inhibitor associated with PLXND1 in HCC.

Transforming growth factor betas (TGF-βs) belong to a family of dimeric peptide growth factors and exists in three isoforms, TGF-β1, TGF-β2, and TGF-β3 (40–42). As a multifunctional cytokine, TGF-β1 was demonstrated to be both a tumor inhibitor, wherein it plays growth inhibitory and anti-inflammatory roles in the early stages of cancer; and a tumor promoter, wherein it promotes the development of aggressive growth characteristics and metastatic spread in late stages of cancer (43, 44). TGF-β1 is also involved in the cancer immune microenvironment and promotion of tumor progression (45). In cancers, especially HCC, immune responses and their modulation play critical roles on the outcome of liver malignancies (46–48). TGF-β1-induced epithelial-mesenchymal transition (EMT) was reported to maintain CD4+ CD25+ regulatory T cells (Tregs) and thus promote HCC invasion and metastasis (49). Interestingly, the present study found that TGF-β1 was the most likely immune inhibitor associated with PLXND1 in HCC. Thus, we further analyzed the patient samples and explored the value of the coexpression of PLXND1 and TGF-β1 as a marker for prognosis. We subclassified liver tumors based on PLXND1 and TGF-β1 expression, and the results showed that this classification could be used to predict clinical outcomes and patient prognosis. Survival analysis showed that the patients with double negative expression (PLXND1^neg^/TGF-β1^neg^) had the best outcome in HCC: H=however, patients with double positive expression (PLXND1^pos^/TGF-β1^pos^) had the worst survival prognosis in HCC. Both univariate and multivariate analyses revealed that the expression of PLXND1 and TGF-β1 could behave as prognostic predictors for both OS and RFS. These findings could be a step toward the development of new biomarkers and therapeutic approaches in HCC.

Nevertheless, several limitations in this study need to be noted. First, the PLXND1 mRNA expression data was extracted from the TCGA database, where ethnicities were mainly white and black, and thus more studies including other ethnic groups should be performed. Additionally, several attractive findings were revealed in this study, but more mechanistic experiments remain to be conducted to further validate the functional value of PLXND1 and TGF-β1 expression in HCC, these aspects are currently under investigation in our lab. Moreover, we mainly focused our analysis on the clinic value of PLXND1 and TGF-β1 for HCC. The analysis of PLXND1 and TGF-β1expression should be further investigated across multiple cancer types.

In conclusion, we first report that PLXND1 is overexpressed in HCC and shows a significant correlation with several clinicopathological features, including tumor stage and TH. Moreover, PLXND1 expression is related with tumor-infiltrating lymphocytes and immunoinhibitors, and TGF-β1 displays the greatest correlations with PLXND1 expression in HCC. Moreover, we further reveal that PLXND1 and TGF-β1 expression are associated with patient prognosis. Taken together, these findings provide a critical understanding of PLXND1 deregulation in HCC and reveal that PLXND1 likely displays an important role in the tumor immune microenvironment. Finally, the results show that PLXNDI is a promising prognostic biomarker in HCC.

## Data Availability Statement

The original contributions presented in the study are included in the article/**Supplementary Material**. Further inquiries can be directed to the corresponding author.

## Ethics Statement

The studies involving human participants were reviewed and approved by the Ethics Review Committee of Xiangya Hospital of Central South University (N201303033). The patients/participants provided their written informed consent to participate in this study.

## Author Contributions

Conception and design: YT and ZW. Writing, review, and/or revision of the manuscript: JL, KH, YT, and ZW. Administrative, technical, or material support: JL, LZ, and DH. All authors contributed to the article and approved the submitted version.

## Funding

This study is supported by grants from the Natural Science Foundation of Hunan Province (2018JJ3820, 12JJ3118) and the National Natural Science Foundation of China (81372630).

## Conflict of Interest

The authors declare that the research was conducted in the absence of any commercial or financial relationships that could be construed as a potential conflict of interest.

## References

[1] Caruso S O’Brien DR Cleary SP Roberts LR Zucman-Rossi J Genetics of HCC: Novel approaches to explore molecular diversity. Hepatology (2020). 10.1002/hep.31394 32463918

[2] Binnewies M Roberts EW Kersten K Chan V Fearon DF Merad M Understanding the tumor immune microenvironment (TIME) for effective therapy. Nat Med (2018) 24(5):541–50. 10.1038/s41591-018-0014-x PMC5998822 29686425

[3] Fu Y Liu S Zeng S Shen H From bench to bed: the tumor immune microenvironment and current immunotherapeutic strategies for hepatocellular carcinoma. J Exp Clin Cancer Res (2019) 38(1):396. 10.1186/s13046-019-1396-4 31500650 PMC6734524

[4] Hu B Lin JZ Yang XB Sang XT The roles of mutated SWI/SNF complexes in the initiation and development of hepatocellular carcinoma and its regulatory effect on the immune system: A review. Cell Prolif (2020) 53(4):e12791. 10.1111/cpr.12791 32162380 PMC7162795

[5] Qiu MJ He XX Bi NR Wang MM Xiong ZF Yang SL Effects of liver-targeted drugs on expression of immune-related proteins in hepatocellular carcinoma cells. Clin Chim Acta (2018) 485:103–5. 10.1016/j.cca.2018.06.032 29940148

[6] Duffy MJ Crown J Biomarkers for Predicting Response to Immunotherapy with Immune Checkpoint Inhibitors in Cancer Patients. Clin Chem (2019) 65(10):1228–38. 10.1373/clinchem.2019.303644 31315901

[7] Vivekanadhan S Mukhopadhyay D Divergent roles of Plexin D1 in cancer. Biochim Biophys Acta Rev Cancer (2019) 1872(1):103–10. 10.1016/j.bbcan.2019.05.004 PMC6692237 31152824

[8] Kong Y Janssen BJ Malinauskas T Vangoor VR Coles CH Kaufmann R Structural Basis for Plexin Activation and Regulation. Neuron (2016) 91(3):548–60. 10.1016/j.neuron.2016.06.018 PMC4980550 27397516

[9] Gay CM Zygmunt T Torres-Vazquez J Diverse functions for the semaphorin receptor PlexinD1 in development and disease. Dev Biol (2011) 349(1):1–19. 10.1016/j.ydbio.2010.09.008 20880496 PMC2993764

[10] Luchino J Hocine M Amoureux MC Gibert B Bernet A Royet A Semaphorin 3E suppresses tumor cell death triggered by the plexin D1 dependence receptor in metastatic breast cancers. Cancer Cell (2013) 24(5):673–85. 10.1016/j.ccr.2013.09.010 24139859

[11] Casazza A Finisguerra V Capparuccia L Camperi A Swiercz JM Rizzolio S Sema3E-Plexin D1 signaling drives human cancer cell invasiveness and metastatic spreading in mice. J Clin Invest (2010) 120(8):2684–98. 10.1172/JCI42118 PMC2912191 20664171

[12] Tseng CH Murray KD Jou MF Hsu SM Cheng HJ Huang PH Sema3E/plexin-D1 mediated epithelial-to-mesenchymal transition in ovarian endometrioid cancer. PLoS One (2011) 6(4):e19396. 10.1371/journal.pone.0019396 21559368 PMC3084850

[13] Mumblat Y Kessler O Ilan N Neufeld G Full-Length Semaphorin-3C Is an Inhibitor of Tumor Lymphangiogenesis and Metastasis. Cancer Res (2015) 75(11):2177–86. 10.1158/0008-5472.CAN-14-2464 25808871

[14] Holl EK O’Connor BP Holl TM Roney KE Zimmermann AG Jha S Plexin-D1 is a novel regulator of germinal centers and humoral immune responses. J Immunol (2011) 186(10):5603–11. 10.4049/jimmunol.1003464 PMC3771081 21464091

[15] Lu C Rong D Zhang B Zheng W Wang X Chen Z Current perspectives on the immunosuppressive tumor microenvironment in hepatocellular carcinoma: challenges and opportunities. Mol Cancer (2019) 18(1):130. 10.1186/s12943-019-1047-6 31464625 PMC6714090

[16] Greten TF Wang XW Korangy F Current concepts of immune based treatments for patients with HCC: from basic science to novel treatment approaches. Gut (2015) 64(5):842–8. 10.1136/gutjnl-2014-307990 PMC6311419 25666193

[17] Hodi FS O’Day SJ McDermott DF Weber RW Sosman JA Haanen JB Improved survival with ipilimumab in patients with metastatic melanoma. N Engl J Med (2010) 363(8):711–23. 10.1056/NEJMoa1003466 PMC3549297 20525992

[18] Lim CJ Lee YH Pan L Lai L Chua C Wasser M Multidimensional analyses reveal distinct immune microenvironment in hepatitis B virus-related hepatocellular carcinoma. Gut (2019) 68(5):916–27. 10.1136/gutjnl-2018-316510 29970455

[19] Li J Alvero AB Nuti S Tedja R Roberts CM Pitruzzello M CBX7 binds the E-box to inhibit TWIST-1 function and inhibit tumorigenicity and metastatic potential. Oncogene (2020) 39(20):3965–79. 10.1038/s41388-020-1269-5 PMC8343988 32205869

[20] Tang Z Li C Kang B Gao G Li C Zhang Z GEPIA: a web server for cancer and normal gene expression profiling and interactive analyses. Nucleic Acids Res (2017) 45(W1):W98–102. 10.1093/nar/gkx247 28407145 PMC5570223

[21] Lian Q Wang S Zhang G Wang D Luo G Tang J HCCDB: A Database of Hepatocellular Carcinoma Expression Atlas. Genomics Proteomics Bioinf (2018) 16(4):269–75. 10.1016/j.gpb.2018.07.003 PMC6205074 30266410

[22] Ru B Wong CN Tong Y Zhong JY Zhong SSW Wu WC TISIDB: an integrated repository portal for tumor-immune system interactions. Bioinformatics (2019) 35(20):4200–2. 10.1093/bioinformatics/btz210 30903160

[23] Wang D Wang ZM Zhang S Wu HJ Tao YM Canopy Homolog 2 Expression Predicts Poor Prognosis in Hepatocellular Carcinoma with Tumor Hemorrhage. Cell Physiol Biochem (2018) 50(6):2017–28. 10.1159/000495048 30415246

[24] Hu K Wang ZM Li JN Zhang S Xiao ZF Tao YM CLEC1B Expression and PD-L1 Expression Predict Clinical Outcome in Hepatocellular Carcinoma with Tumor Hemorrhage. Transl Oncol (2018) 11(2):552–8. 10.1016/j.tranon.2018.02.010 PMC5884195 29525632

[25] Oh WJ Gu C The role and mechanism-of-action of Sema3E and Plexin-D1 in vascular and neural development. Semin Cell Dev Biol (2013) 24(3):156–62. 10.1016/j.semcdb.2012.12.001 PMC3612391 23270617

[26] Mehta V Pang KL Rozbesky D Nather K Keen A Lachowski D The guidance receptor plexin D1 is a mechanosensor in endothelial cells. Nature (2020) 578(7794):290–5. 10.1038/s41586-020-1979-4 PMC7025890 32025034

[27] Minchin JE Dahlman I Harvey CJ Mejhert N Singh MK Epstein JA Plexin D1 determines body fat distribution by regulating the type V collagen microenvironment in visceral adipose tissue. Proc Natl Acad Sci USA (2015) 112(14):4363–8. 10.1073/pnas.1416412112 PMC4394244 25831505

[28] Roodink I Kats G van Kempen L Grunberg M Maass C Verrijp K Semaphorin 3E expression correlates inversely with Plexin D1 during tumor progression. Am J Pathol (2008) 173(6):1873–81. 10.2353/ajpath.2008.080136 PMC2626397 18974298

[29] Roodink I Raats J van der Zwaag B Verrijp K Kusters B van Bokhoven H Plexin D1 expression is induced on tumor vasculature and tumor cells: a novel target for diagnosis and therapy? Cancer Res (2005) 65(18):8317–23. 10.1158/0008-5472.CAN-04-4366 16166308

[30] Roodink I Verrijp K Raats J Leenders WP Plexin D1 is ubiquitously expressed on tumor vessels and tumor cells in solid malignancies. BMC Cancer (2009) 9:297. 10.1186/1471-2407-9-297 19703316 PMC2739226

[31] Huang HY Cheng YY Liao WC Tien YW Yang CH Hsu SM SOX4 transcriptionally regulates multiple SEMA3/plexin family members and promotes tumor growth in pancreatic cancer. PLoS One (2012) 7(12):e48637. 10.1371/journal.pone.0048637 23251334 PMC3520963

[32] Yan Y Liu W Liu M Gong Z Xu Z Immune Cell Infiltration Influences Long-Term Survivorship of Patients with SCLC. J Thorac Oncol (2019) 14(10):e241. 10.1016/j.jtho.2019.06.017 31558241

[33] Sun J Zhang Z Bao S Yan C Hou P Wu N Identification of tumor immune infiltration-associated lncRNAs for improving prognosis and immunotherapy response of patients with non-small cell lung cancer. J Immunother Cancer (2020) 8(1):e000110. 10.1136/jitc-2019-000110 PMC7057423 32041817

[34] Liu R Hu R Zeng Y Zhang W Zhou HH Tumour immune cell infiltration and survival after platinum-based chemotherapy in high-grade serous ovarian cancer subtypes: A gene expression-based computational study. EBioMedicine (2020) 51:102602. 10.1016/j.ebiom.2019.102602 31911269 PMC6948169

[35] Vinuesa CG Linterman MA Yu D MacLennan IC Follicular Helper T Cells. Annu Rev Immunol (2016) 34:335–68. 10.1146/annurev-immunol-041015-055605 26907215

[36] Kawakami T Koike A Amano F Sodium bicarbonate regulates nitric oxide production in mouse macrophage cell lines stimulated with lipopolysaccharide and interferon gamma. Nitric Oxide (2018) 79:45–50. 10.1016/j.niox.2018.07.008 30063984

[37] Xiao J Xie R Li Q Chen W Zhang Y Generation and characterization of bovine bone marrow-derived macrophage cell line. Cell Biol Int (2016) 40(5):603–8. 10.1002/cbin.10599 26936441

[38] Lv Y Zhao Y Wang X Chen N Mao F Teng Y Increased intratumoral mast cells foster immune suppression and gastric cancer progression through TNF-alpha-PD-L1 pathway. J Immunother Cancer (2019) 7(1):54. 10.1186/s40425-019-0530-3 30808413 PMC6390584

[39] Aponte-Lopez A Fuentes-Panana EM Cortes-Munoz D Munoz-Cruz S Mast Cell, the Neglected Member of the Tumor Microenvironment: Role in Breast Cancer. J Immunol Res (2018) 2018:2584243. 10.1155/2018/2584243 29651440 PMC5832101

[40] Batlle E Massague J Transforming Growth Factor-beta Signaling in Immunity and Cancer. Immunity (2019) 50(4):924–40. 10.1016/j.immuni.2019.03.024 PMC7507121 30995507

[41] Meng XM Nikolic-Paterson DJ Lan HY TGF-beta: the master regulator of fibrosis. Nat Rev Nephrol (2016) 12(6):325–38. 10.1038/nrneph.2016.48 27108839

[42] Achyut BR Yang L Transforming growth factor-beta in the gastrointestinal and hepatic tumor microenvironment. Gastroenterology (2011) 141(4):1167–78. 10.1053/j.gastro.2011.07.048 PMC6644047 21839702

[43] Zhu H Luo H Shen Z Hu X Sun L Zhu X Transforming growth factor-beta1 in carcinogenesis, progression, and therapy in cervical cancer. Tumour Biol (2016) 37(6):7075–83. 10.1007/s13277-016-5028-8 27010470

[44] Tang J Gifford CC Samarakoon R Higgins PJ Deregulation of Negative Controls on TGF-beta1 Signaling in Tumor Progression. Cancers (Basel) (2018) 10(6). 10.3390/cancers10060159 PMC6025439 29799477

[45] Zarzynska JM Two faces of TGF-beta1 in breast cancer. Mediators Inflamm (2014) 2014:141747. 10.1155/2014/141747 24891760 PMC4033515

[46] Eggert T Wolter K Ji J Ma C Yevsa T Klotz S Distinct Functions of Senescence-Associated Immune Responses in Liver Tumor Surveillance and Tumor Progression. Cancer Cell (2016) 30(4):533–47. 10.1016/j.ccell.2016.09.003 PMC7789819 27728804

[47] Qasim W Brunetto M Gehring AJ Xue SA Schurich A Khakpoor A Immunotherapy of HCC metastases with autologous T cell receptor redirected T cells, targeting HBsAg in a liver transplant patient. J Hepatol (2015) 62(2):486–91. 10.1016/j.jhep.2014.10.001 25308176

[48] Inada Y Mizukoshi E Seike T Tamai T Iida N Kitahara M Characteristics of Immune Response to Tumor-Associated Antigens and Immune Cell Profile in Patients With Hepatocellular Carcinoma. Hepatology (2019) 69(2):653–65. 10.1002/hep.30212 30102778

[49] Shi C Chen Y Chen Y Yang Y Bing W Qi J CD4(+) CD25(+) regulatory T cells promote hepatocellular carcinoma invasion via TGF-beta1-induced epithelial-mesenchymal transition. Onco Targets Ther (2019) 12:279–89. 10.2147/OTT.S172417 PMC6314313 30643426

