# Supplementary material for: Prognostic Value of PLXND1 and TGF-β1 Coexpression and Its Correlation With Immune Infiltrates in Hepatocellular Carcinoma
Source: Front Oncol. 2021 Jan 8;10:604131. doi: 10.3389/fonc.2020.604131 (PMC7820679; doi:10.3389/fonc.2020.604131)
Supplement: Supplementary file 1 [file DataSheet_1.docx]

**
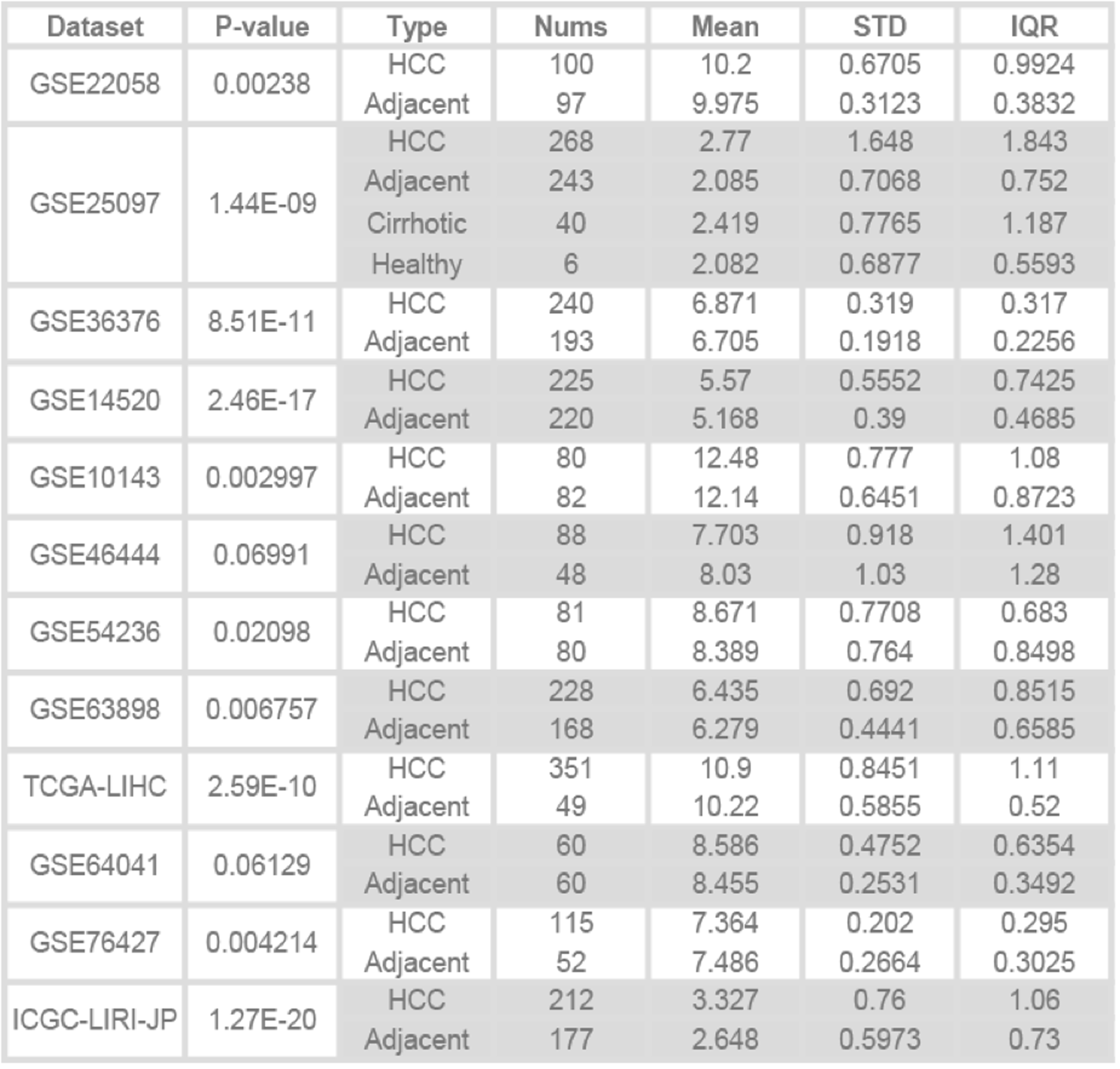
**

**Supplementary Figure 1. PLXND1 expression analysis in HCCDB.** PLXND1 expression in 10 out of 12 clinical cohorts was significantly different between HCC and adjacent tissues.

**
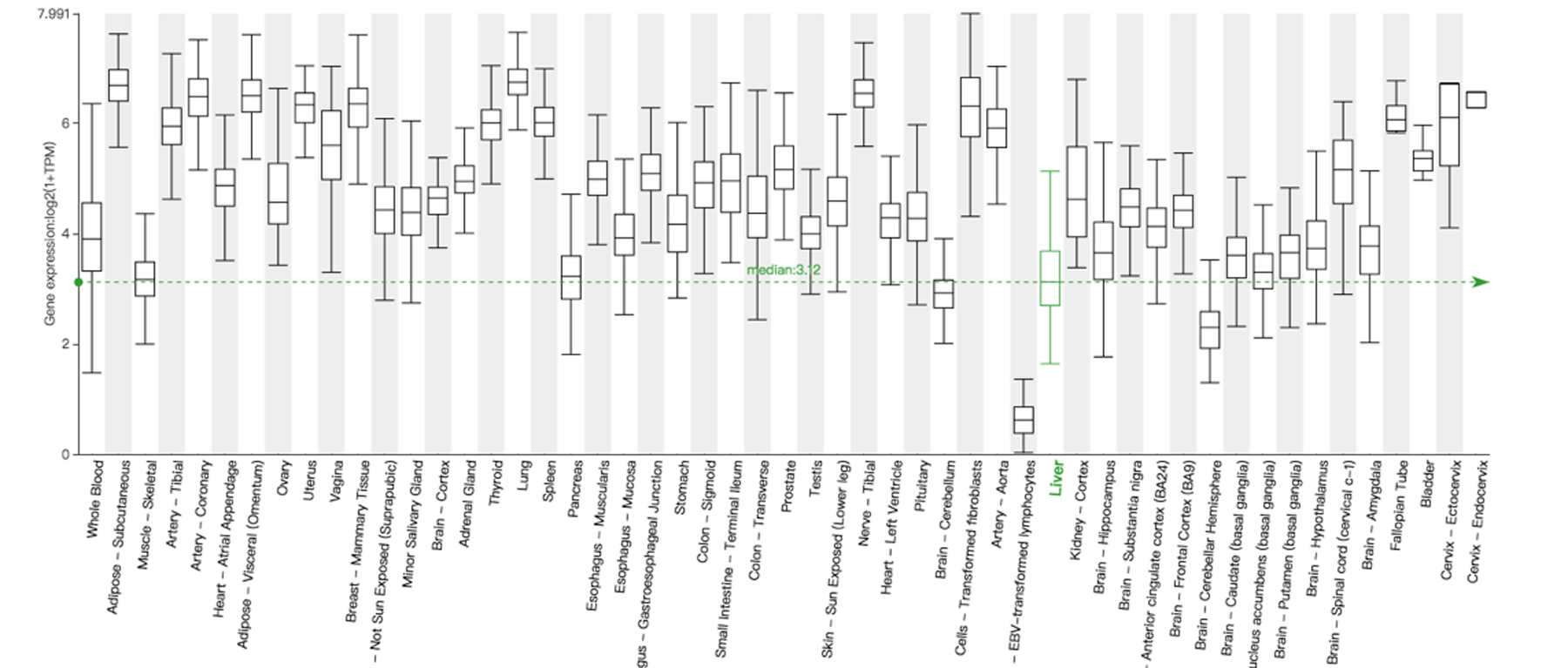
**

**Supplementary Figure 2. PLXND1 expression in normal tissues.** PLXND1 expression varied among 53 types of normal tissues, and its expression in normal liver tissue was very low.


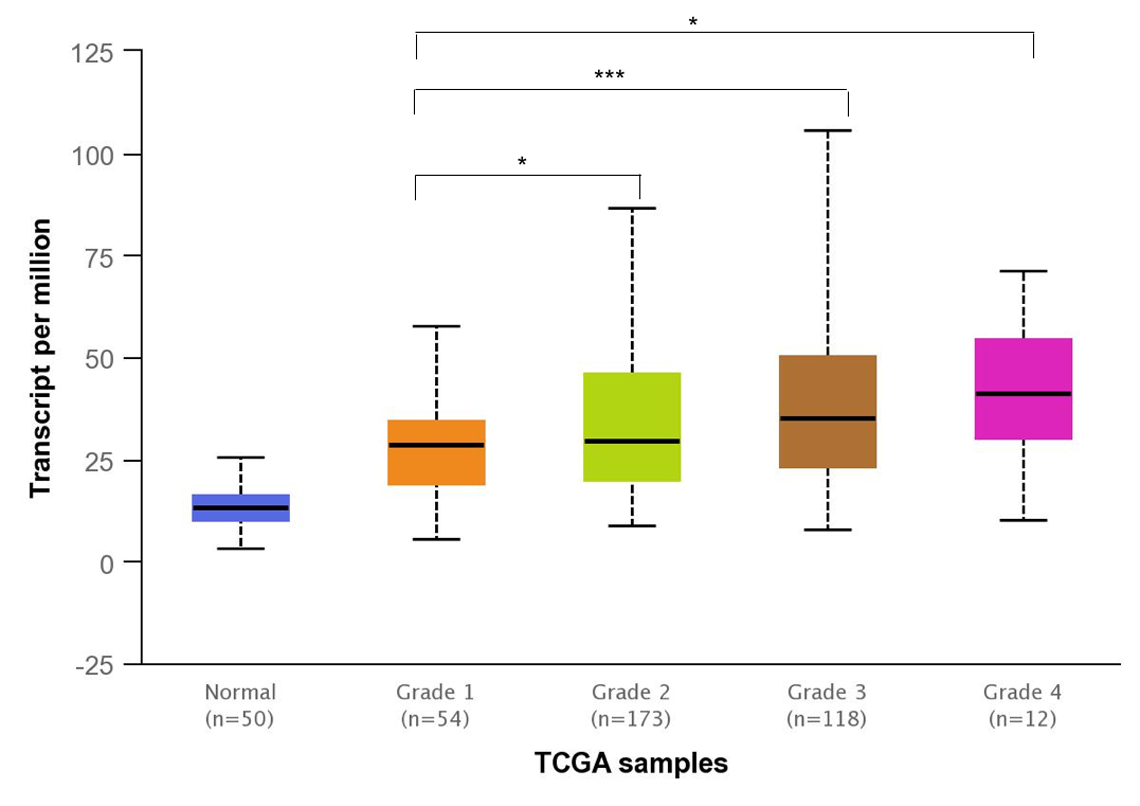


**Supplementary Figure 3. PLXND1 expression levels at in different HCC stages from UALCAN.** The expression of PLXND1 was significantly higher in higher-grade HCC tissues. A boxplot shows the relative expression of PLXND1 in normal individuals or HCC patients with grade 1, 2, 3 or 4 tumors. The central mark is the median; the edges of the box are the 25th and 75th percentiles. *, p < 0.05; ***, p < 0.001.

**Supplementary Table S1. Correlation analysis between PLXND1 and markers of immune infiltrating cells via GEPIA.**

| Description | Gene | HCC | | | | |
| --- | --- | --- | --- | --- | --- | --- |
|  |  | Tumor | |  | Normal liver | |
|  |  | R | P |  | R | P |
| Treg | TGF-β1 | 0.43 | *** |  | 0.82 | *** |
|  | FOXP3 | -0.007 | 0.16 |  | 0.03 | 0.8 |
|  | STAT5B | -0.001 | 0.75 |  | 0.61 | *** |
|  | CCR8 | 0.11 | 0.043 |  | 0.60 | *** |
| CD4+ T cell | CD27 | 0.24 | *** |  | 0.70 | *** |
|  | CD86 | 0.43 | *** |  | 0.78 | *** |
| CD8+ T cell | CD8A | 0.26 | *** |  | 0.67 | *** |
|  | CD8B | 0.24 | *** |  | 0.73 | *** |
| T cell (general) | CD2 | 0.23 | *** |  | 0.69 | *** |
|  | CD3D | 0.19 | *** |  | 0.70 | *** |
|  | CD3E | 0.22 | *** |  | 0.73 | *** |
| B cell | CD19 | 0.02 | 0.63 |  | 0.48 | *** |
|  | CD79A | 0.03 | 0.46 |  | 0.55 | *** |
| Monocyte | CD86 | 0.43 | *** |  | 0.78 | *** |
|  | CD115 (CSF1R) | 0.45 | *** |  | 0.76 | *** |
| M1 Macrophage | INOS (NOS2) | -0.02 | 0.70 |  | 0.16 | 0.26 |
|  | IRF5 | 0.2 | *** |  | 0.74 | *** |
|  | COX2 (PTGS2) | 0.06 | 0.23 |  | 0.24 | 0.09 |
| M2 Macrophage | CD163 | 0.48 | *** |  | 0.69 | *** |
|  | VSIG4 | 0.45 | *** |  | 0.57 | *** |
|  | MS4A4A | 0.45 | *** |  | 0.77 | *** |
| TAM | CCL2 | 0.12 | *** |  | 0.33 | 0.01 |
|  | CD68 | 0.26 | *** |  | 0.75 | *** |
|  | IL10 | 0.62 | *** |  | 0.45 | *** |
| Natural killer cell | KIR2DL1 | 0.008 | 0.88 |  | 0.12 | 0.40 |
|  | KIR2DL3 | 0.096 | 0.067 |  | 0.13 | 0.48 |
|  | KIR2DL4 | 0.13 | 0.01 |  | 0.18 | 0.22 |
|  | KIR3DL1 | 0.01 | 0.72 |  | 0.10 | 0.48 |
|  | KIR3DL2 | 0.04 | 0.45 |  | -0.02 | 0.84 |
|  | KIR3DL3 | 0.001 | 0.98 |  | 0.07 | 0.60 |
|  | KIR2DS4 | 0.073 | 0.16 |  | 0.006 | 0.96 |
|  | KLRK1 (NKG2D) | 0.20 | *** |  | 0.72 | *** |
|  | NCR1 (NKp46) | 0.12 | 0.025 |  | 0.3 | 0.033 |
|  | NCR2 (NKp44) | 0.03 | 0.54 |  | 0.29 | 0.041 |
|  | NCR3 (NKp30) | 0.14 | *** |  | 0.76 | *** |
| Dendritic cell | HLA-DPB1 | 0.34 | *** |  | 0.83 | *** |
|  | HLA-DQB1 | 0.29 | *** |  | 0.36 | *** |
|  | HLA-DRA | 0.31 | *** |  | 0.79 | *** |
|  | BDCA1 (CD1C) | 0.18 | *** |  | 0.56 | *** |
|  | BDCA4 (NRP1) | 0.23 | 0.11 |  | 0.34 | *** |
|  | CD11c (ITGAX) | 0.22 | *** |  | 0.60 | *** |
| Neutrophils | CD66b (CEACAM8) | 0.01 | 0.77 |  | 0.16 | 0.25 |
|  | CD11b(ITGAM) | 0.18 | *** |  | 0.68 | *** |
|  | CCR7 | 0.05 | 0.27 |  | 0.65 | *** |
| T-helper 1 cell | T-bet (TBX21) | 0.15 | *** |  | 0.79 | *** |
|  | STAT4 | 0.13 | 0.01 |  | 0.68 | *** |
|  | TNF-α | 0.048 | 0.35 |  | 0.52 | *** |
| T-helper 2 cell | GATA3 | 0.31 | *** |  | 0.24 | 0.09 |
|  | STAT6 | 0.004 | 0.42 |  | 0.66 | *** |
|  | STAT5A | 0.50 | *** |  | 0.80 | *** |
|  | IL13 | -0.03 | 0.51 |  | -0.05 | 0.70 |
|  | IL21 | -0.017 | 0.75 |  | 0.19 | 0.20 |
| T-helper 17 cell | IL17A | -0.044 | 0.40 |  | 0.097 | 0.50 |
|  | STAT3 | 0.059 | 0.26 |  | -0.043 | 0.77 |
| T cell exhaustion | PD-1 (PDCD1) | 0.17 | *** |  | 0.57 | *** |
|  | CTLA4 | 0.16 | *** |  | 0.56 | *** |
|  | LAG3 | 0.25 | *** |  | 0.43 | *** |
|  | TIM-3 | 0.30 | *** |  | 0.65 | *** |
| Mast cells | TPSB2 | 0.22 | *** |  | 0.04 | 0.74 |
|  | TPSAB1 | 0.25 | *** |  | 0.35 | 0.01 |
|  | CPA3 | 0.13 | *** |  | 0.29 | 0.04 |
|  | MS4A2 (HAVCR2) | 0.21 | *** |  | 0.41 | *** |
|  | HDC | 0.074 | 0.15 |  | -0.16 | 0.25 |
